# Risks and Complications of Coronary Angiography: A Comprehensive Review

**DOI:** 10.5539/gjhs.v4n1p65

**Published:** 2012-01-01

**Authors:** Morteza Tavakol, Salman Ashraf, Sorin J. Brener

**Affiliations:** New York Methodist Hospital United States of America

**Keywords:** Cardiac catheterization, Angiography, Contrast material, Acute kidney injury, Complications

## Abstract

Coronary angiography and heart catheterization are invaluable tests for the detection and quantification of coronary artery disease, identification of valvular and other structural abnormalities, and measurement of hemodynamic parameters. The risks and complications associated with these procedures relate to the patient’s concomitant conditions and to the skill and judgment of the operator. In this review, we examine in detail the major complications associated with invasive cardiac procedures and provide the reader with a comprehensive bibliography for advanced reading.

## 1. Introduction

Coronary angiography is the gold standard test for identifying the presence and extent of atherosclerotic coronary artery disease (CAD). As with any invasive procedure, there are specific patient-dependent and procedure-related complications that are inherent to the test. Complications range widely from minor problems with short term sequelae to life threatening situations that may cause irreversible damage, if urgent care is not provided. Fortunately, the associated risks have decreased significantly since the inception of coronary arteriography due to advanced equipment design, improved peri-procedural management, and increased experience of diagnostic centers and operators.

Although there are no absolute contraindications to performing coronary arteriography, the associated risks can be attributed to cardiac and non-cardiac complications. Specific disease states pertaining to the general medical profile of the patient (older age, renal insufficiency, uncontrolled diabetes mellitus, and morbid obesity) can increase the risk for complications. The underlying cardiovascular status of the patient can further predispose to adverse events. The extent of CAD, congestive heart failure (CHF) with low ejection fraction, recent stroke or myocardial infarction (MI), and bleeding propensity are just a few of the cardiovascular characteristics that can increase cardiac and vascular complications. Furthermore, the type of procedure being performed, be it diagnostic coronary angiography or additional percutaneous coronary intervention, modulates the risk.

Given the above considerations, however, major complications are uncommon. Because major complications from cardiac catheterization occur in less than 2% of the population, with mortality of less than 0.08%, there are relatively few patients who cannot be studied safely in an experienced laboratory. The use of iso-osmolar contrast media, lower profile diagnostic catheters, measures to reduce the incidence of bleeding and extensive operator experience can all serve to reduce the already low incidence of such complications even further. Therefore, the procedure can be successfully performed even in the most critically ill patient, when clinically indicated, with relatively low risk. However, the risk-to-benefit ratio of cardiac catheterization and familiarity with potential benefits and risks must be assessed on an individual basis in order to minimize any potential problems. In this chapter, we aim to identify the risks associated with coronary angiography and coronary interventions in the modern catheterization laboratory, and describe advances in equipment design and management protocols that have been promoted to reduce potential complications.

## 2. Allergic and Adverse Reactions

### 2.1 Local Anesthesia

Allergic local and systemic reactions to local anesthesia are extremely rare. Methemglobinemia, asthma-like reactions, vasodepressor reaction and anesthesia toxicity have been reported (Finder & Moore, 2002). Most reports are with the older agents and have been infrequently reported with amide agents, such as lidocaine. Reactions are generally dermatologic or vagal, and are rarely anaphylactic. The reactions that do occur are generally secondary to the preservatives used in drug preparations. Use of preservative-free agents, such as bupivacaine, and skin testing would be warranted in patients with history of reactions to local anesthetics (T. Feldman, Moss, Teplinsky, & Carroll, 1990).

### 2.2 General Anesthesia

General anesthesia is not routinely required in the catheterization laboratory, and the vast majority of procedures occur without the presence of an anesthesiologist. Conscious sedation and analgesia with short acting agents such as midazolam or fentanyl at low doses are, however, commonly used to increase patient comfort and relieve anxiety during the procedure. In such cases, care must be taken to avoid over-sedation of the patient. Close monitoring of blood pressure, heart rate, respiratory rate and oxygenation should be performed in all patients. When hemodynamic compromise or oversedation occurs, the use of reversal agents for benzodiazepines (flumazenil) and opiates (naloxone) should be promptly administered. Anaphylactoid reactions happen infrequently with conscious sedation agents and are much more likely to occur following administration of contrast media. Treatment of any adverse reaction depends on its severity, and includes the potential use of oxygen, bronchodilators, epinephrine, histamine blockers, corticosteroids and intravenous fluids (Dewachter, Mouton-Faivre, & Emala, 2009). In cases of severe anaphylaxis not responsive to conservative management, endotracheal intubation and urgent consultation with anesthesia team must be performed. Proper history and review of allergies can help avoid unnecessary exposure to patients with previous allergy or adverse reactions to local or systemic anesthesia. Particular attention should be paid to allergy to sea food, as there is possible cross-reactivity with iodine-containing contrast media.

### 2.3 Contrast Media

Adverse reactions from contrast media may be classified as chemotoxic or anaphylactoid. Contrast media stimulate an anaphylactoid response through histamine release. It differs from an anaphylactic reaction, in that it is not immune-mediated and does not require prior sensitization to the offending agent to initiate a reaction. Chemotoxic effects are primarily related to the hyperosmolarity, ionic content, viscosity, and calcium binding properties of these agents (Goss, Chambers, & Heupler, 1995). All contrast agents are based exclusively on iodine, commonly combined to a benzoic acid ring in a mixture of meglumine or sodium salt of diatrizoid acid with calcium EDTA. The concentrations of sodium and EDTA are kept roughly equal to that of blood, as higher or lower concentrations have been associated with tachyarrhythmia and myocardial depression. In order to achieve the iodine concentration that is needed for optimal visualization during angiography, solutions of conventional contrast agents were extremely hypertonic. The resulting solutions of these agents (Hypaque (Nycomed) and Angiovist (Berlex)) have an osmolality about 5.8 times (1690 mOsm/kg) that of plasma (Barrett *et al.*, 1992). Adverse reactions are common in the ionic, high osmolality contrast agents, reported in > 50% of patients in some studies (Matthai *et al.*, 1994). Milder constitutional symptoms are frequently reported (warmth, pain, chest tightness, nausea and vomiting) and are self-limited in the majority of cases. Adverse reactions requiring intervention (hypotension, bradyarrhythmias, pulmonary congestion) have been reported in nearly 30% of patients in one randomized trial (Barrett *et al.*, 1992).

The introduction of lower osmolar, ionic agents (ioxaglate (Hexabrix)), and water soluble low-osmolar, non-ionic (iohexol (Omnipaque), ioxilan (Oxilan)) have significantly reduced the incidence of hypersensitivity and adverse reactions. In randomized clinical trials, the use of high osmolar contrast material was associated with a 3.1% increase in risk for need to treat patients for adverse reactions and 3.6% increase in life threatening reactions in comparison to use of lower osmolar non-ionic agents. These reactions were largely confined to patients with severe coronary artery disease or unstable angina (Barrett *et al.*, 1992). These results have been duplicated in two other randomized trials that were able to further risk stratify patients at highest risk for developing adverse contrast reactions (Matthai *et al.*, 1994; Steinberg *et al.*, 1992). Patients with advanced age, higher New York Heart Association CHF class, history of prior contrast reaction, and elevated left ventricular diastolic pressure have been identified as being up to six times more likely to develop adverse reactions with high osmolar ionic agents (Matthai *et al.*, 1994). The need for risk stratification initially arose from the high cost of the newer low osmolar agents, which was at one point 10-20 times that of conventional high osmolar agents (Barrett *et al.*, 1992). Selective use of these agents in appropriate populations has been shown to decrease overall cost by 66% with improvement in safety and cost-effectiveness (Matthai *et al.*, 1994). The cost of these agents, however, has decreased significantly over the past 10 years allowing for more widespread use of the low osmolar agents to prevent adverse reactions at only a small incremental difference in price.

Most recently, a non-ionic, iso-osmolar compound (iodixanol (Visipaque) has been developed that has an osmolality similar to that of blood (290 mOsm/kg). Hypersensitivity reactions occurred in only 0.7% of the population studied in a large randomized trial comparing iodixanol to the ionic, low osmolar agent ioxaglate, without a significant difference in major cardiovascular events (Bertrand, Esplugas, Piessens, & Rasch, 2000). The introduction of the non-ionic agents was initially met with some concern due to evidence that ionic contrast material exhibited a more pronounced antiplatelet and antithrombotic activity, especially in *in-vitro* studies. These properties may be beneficial during a procedure that may damage the vascular endothelium and cause thrombosis. Fortunately, no increase in the risk of thrombotic complications or major cardiovascular events has been demonstrated in large randomized multicenter trials of angioplasty in which the two classes of contrast agents were compared (Bertrand *et al.*, 2000; Schrader *et al.*, 1999)

### 2.4 Prophylaxis and Treatment

Prevention of allergic reactions to contrast material can be successfully achieved. There are two categories of patients at risk for developing anaphylaxis that should be considered for pre-treatment. Patients with previous anaphylactic reactions are at highest risk for developing recurrent reactions. The second category consists of patients with history of atopy, asthma or those who take beta adrenergic blockers, in whom a twofold risk in anaphylaxis has been reported (Lang, Alpern, Visintainer, & Smith, 1991). Despite general concerns, no consistent cross-reactivity has been demonstrated in patients with allergies to food containing iodine (seafood) and contrast anaphylaxis risk (Goss *et al.*, 1995; Hildreth, 1987). When encountering patients with history of allergy to shellfish or seafood, further questioning should be addressed toward history of atopy or asthma, as this would identify the patients at highest risk for developing anaphylaxis. In addition to the type of contrast agent, pre-treatment with prophylactic medications is a critical part of preventing recurrent reactions in the population at highest risk. Corticosteroids and histamine blockers are the cornerstone of pretreatment. Prednisone 50 mg administered 13, 7, and 1 hour before the procedure together with diphenhydramine 50 mg orally 1 hour before the procedure are effective in reducing recurrent reactions (Bush & Swanson, 1991; Goss *et al.*, 1995; Greenberger, Halwig, Patterson, & Wallemark, 1986; Nayak, White, Cavendish, Barker, & Kandzari, 2009; Wittbrodt & Spinler, 1994). For urgent procedures, intravenous hydrocortisone 200 mg with 50 mg of diphenhydramine should be used prior to the procedure (Table 1) (Greenberger *et al.*, 1986).

**Table 1:** Specific recommendation for pre-medication regimens. Adapted from the American College of Radiology guidelines (Amreican College of Radiology, 2010). Note that use of H2 blockers is not supported by the current guidelines.

Elective Pre-Medication	1. Prednisone 50 mg by mouth at 13 hours, 7 hours, and 1 hour before contrast media injection 2. Diphenhydramine 50 mg intravenous, intramuscular, or by mouth 1 hour before contrast medium injection
Emergency Pre-Medication (Decreasing order of desirability)	1. Methylprednisolone 40 mg or hydrocortisone sodium succinate 200 mg intravenously every 4 hours until contrast study required plus diphenhydramine 50 mg intravenous 1 hour prior to contrast injection 2. Dexamethasone sodium sulfate 7.5 mg or betamethasone 6.0 mg every 4 hours until contrast study. Must be done in patients with known allergy to methylprednisolone, aspirin, or nonsteroidal anti-inflammatory drugs, especially if asthmatic. Also diphenhydramine 50 mg intravenous 1 hour prior to contrast injection. 3. Omit steroids entirely and give diphenhydramine 50 mg intravenous.

It has been hypothesized that the addition of Histamine-2 blockers (cimetidine or ranitidine) to the above regimen may provide greater antihistamine effect on the vascular system in addition to diphenhydramine, a conventional Histamine-1 blocker. The low cost and high safety profile of Histamine-2 blockers have made them a common component of treatment in many catheterization laboratories. Its effectiveness, however, is controversial, and consistent results have not been shown in prospective trials (Goss *et al.*, 1995; Greenberger *et al.*, 1986; Myers & Bloom, 1981; Wittbrodt & Spinler, 1994). Monteleukast has also been advocated as therapeutic addition. The use of Histamine-2 blockers and Monteleukast has not been advocated by the American College of Radiology (American College of Radiology, 2010).

Despite adequate pre-medication, breakthrough reactions have been shown to occur (Freed, Leder, Alexander, DeLong, & Kliewer, 2001), stressing the role of awareness and careful monitoring in this group of patients. In the case of anaphylactic reactions with laryngeal edema and vascular compromise, 0.3 ml epinephrine at a dilution of 1:1000 subcutaneously or 3 ml at dilution of 1:10,000 intravenously or subcutaneously should be administered immediately. Corticosteroids, diphenhydramine and large volume intravenous fluids should also be given to decrease the severity of the reaction. The use of Histamine-2 blockers remains controversial but should be considered in treatment of refractory cases (Bush & Swanson, 1991; Goss *et al.*, 1995).

### 2.5 Heparin Induced Thrombocytopenia

Heparin Induced Thrombocytopenia (HIT) is a serious immune-mediated complication of heparin administration from flush heparinized saline or during percutaneous coronary interventions. Although the risk will not be manifest during the procedure, the clinical symptoms that develop in the days after the procedure can have potentially devastating thromboembolic complications in patients with prior exposure to heparin. Roughly 1-3% of patients who receive unfractionated heparin will develop a serious form of immune mediated thrombocytopenia with associated venous and arterial thrombosis (HIT-2) (Brieger, Mak, Kottke-Marchant, & Topol, 1998; Jang & Hursting, 2005). This reaction is caused by antibodies binding to the heparin platelet factor-4 complex, which lead to a cascade of reactions causing platelet activation and the release of procoaggulant and inflammatory factors that consume platelets and incite thrombosis. Patients that develop HIT-2 usually experience a platelet count drop of at least 50%, typically 5-15 days after initiation of heparin, or more suddenly following previous heparin sensitization (Jang & Hursting, 2005). Patients with underlying coronary artery disease and patients with cardiac transplantation have a higher incidence of HIT (2-8% and 11%, respectively) (Hourigan, Walters, Keck, & Dec, 2002; Kappers-Klunne *et al.*, 1997), and several patients have been described in whom an acute coronary syndrome (manifesting as acute thrombosis) occurred during coronary angioplasty in association with the onset of HIT (Gupta, Savage, & Brest, 1995). The diagnosis is based on the clinical picture of platelet decrease with or without associated thrombosis. HIT-antibody assays are routinely available for confirming the diagnosis, but treatment should not be delayed when there is a strong clinical suspicion due to severity of comorbidities. Among the patients with HIT and thrombosis, 9-11% require limb amputation and mortality is reported in 17-30% (Jang & Hursting, 2005). Treatment includes immediate and complete discontinuation of heparin and initiation of treatment with direct thrombin inhibitors, such as argatroban, bivlaurdin, or lepirudin. In patients with, or at risk for HIT, who present to the catheterization laboratory, prospective trials of bivalirudin and argatroban have demonstrated safety and efficacy (Campbell *et al.*, 2000; Lewis *et al.*, 2002; Mahaffey *et al.*, 2003). Bivalirudin dose needs adjustment in patients with severe renal impairment, while argatroban is contraindicated in patients with hepatic dysfunction.

## 3. Infections

### 3.1 Incidence

Infections are rare after invasive cardiovascular procedures. The reported incidence of catheter-related infections (not involving cut-down techniques) is much less than < 1% based on retrospective studies (Munoz *et al.*, 2001; Ramsdale, Aziz, Newall, Palmer, & Jackson, 2004). This may be an underestimation of the true incidence of infections acquired during catheterization, as most signs and symptoms are unlikely to develop immediately following the procedure. In a prospective study of 147 consecutive blood cultures obtained after complex cardiac catheterization procedures, positive blood cultures were found in 18% and 12% of the subjects immediately following and 12 hours after the procedure, respectively. The most common organism was coagulase negative staphylococcus and none of the patients developed clinical signs of infection (Ramsdale *et al.*, 2004).

Fever is a relative contraindication for elective procedures. Patients with ongoing infections should be appropriately treated before an elective cardiac catheterization (Chambers *et al.*, 2006). Certain catheterization techniques have been shown in case studies to increase the risk of infectious complications. Local infections after angioplasty have been related to early re-puncture of ipsilateral femoral artery (Wiener & Ong, 1989), use of arterial grafts for access (McCready *et al.*, 1991), and retention of catheters for prolonged periods (Polanczyk *et al.*, 2001). Local hematomas can be a nidus of infection and should be treated urgently upon occurrence. Infection of the suture or collagen anchor in vascular closure devices are infrequent (0.5%), but can lead to limb-threatening arteritis when they occur (Baddour *et al.*, 2004; Cooper & Miller, 1999). Insertion of a Foley catheter prior to the procedure should be noted as a potential cause of complicated urinary tract infection. Their use should be avoided when possible and, when inserted, removed when urine output monitoring is not further warranted.

### 3.2 Infectious Precautions

The American College of Cardiology does not recommend the use of strict operating room sterile techniques for most catheterization procedures (Bashore *et al.*, 2001; Chambers *et al.*, 2006).

Hair removal should be considered if there is interference with obtaining access site. When removal is necessary, electric clippers should be used and the use of razors should be avoided (Chambers *et al.*, 2006; Ko, Lazenby, Zelano, Isom, & Krieger, 1992; O’Grady *et al.*, 2002). Skin cleansing with a 2% chlorhexadine based preparation such as Chloraprep should be used prior to local anesthesia. A recent study of ~500 patients did not detect any difference in the rate of infections when caps and masks were worn (Laslett & Sabin, 1989). However, studies looking at the effectiveness of sterile techniques in the catheterization laboratory will require a large patient population in order to be sufficiently powered, given the low incidence of infection. Furthermore, the use of masks and eye shields may provide more protection to the operator to avoid blood splattering during the procedure. Following the procedure, the use of occlusive dressings and topical antimicrobials should be avoided as they can increase the risk of bacterial and fungal infections (Chambers *et al.*, 2006). Antibiotic prophylaxis is not routinely indicated during cardiac catheterization (O’Grady *et al.*, 2002).

## 4. Nephropathy

Contrast Induced Nephropathy (CIN) is a potentially serious complication of coronary angiography with significant short and long term sequelae. CIN, however, can be minimized with proper risk stratification, selection of contrast agent and staging of procedures, along with preventive management strategies. CIN has been defined as rise in serum creatinine of ≥ 0.5 mg/dl or 25% above the baseline value, based on data that associated such increases with clinically relevant outcomes, such as permanent renal impairment requiring hemodialysis, and death (Gami & Garovic, 2004). Varying definitions of CIN applied in studies with differences in patient co-morbidity have led to difficulty in assessing the true incidence of CIN, with reported rates ranging between 3.3-16.5% (Murphy, Barrett, & Parfrey, 2000). A large observational study in 1,826 consecutive patients uncovered an incidence of 14.4% in a community based population (McCullough, Wolyn, Rocher, Levin, & O’Neill, 1997). Smaller prospective studies in patients with fewer risk factors have shown a much smaller risk, roughly 3% (Rudnick, Berns, Cohen, & Goldfarb, 1997). Most patients, fortunately, experience a mild, transient increase in serum creatinine that is typically not associated with oliguria, peaks within two to four days, and generally resolves by 7 days.

The pathogenesis of CIN appears to be multifactorial. Multidirectional changes in renal hemodynamics due to the effects of contrast media on a number of vasoactive substances (adenosine, nitric oxide, endothelin) along with direct cytotoxicity through the action of free radicals have been implicated as potential causes (Barrett & Carlisle, 1993; R. Solomon, 2005). Preexisting renal insufficiency, diabetes, age, along with osmolality and volume of contrast used are the most significant risk factors to developing CIN. In retrospective studies of patients undergoing angiography, the incidence of CIN in patients with baseline creatinine < 2.0 mg/dl was higher among diabetic than nondiabetic patients. Amongst those with a baseline creatinine ≥ 2.0, all had a significantly higher risk of acute renal failure. Of the 7,856 patients studied, the risk of CIN was only 2.5% when creatinine was < 2 mg/dl, but rose to 30.6% when creatinine was > 3.0 mg/dl (Rihal *et al.*, 2002). In patients who develop acute renal failure, the two largest studies have reported a nearly identical risk of 7.1% for suffering permanent kidney damage requiring hemodialysis (McCullough, Bertrand, Brinker, & Stacul, 2006; Rihal *et al.*, 2002). In addition, multiple studies have shown a correlation between CIN and poor long-term survival (Bartholomew *et al.*, 2004; Freeman *et al.*, 2002; Rihal *et al.*, 2002) with the risk of renal injury requiring dialysis, recurrent hospitalization, and death increasing proportionally to the severity of acute kidney injury (James *et al*.). In large registries, 22% of patients with acute renal failure die during the index hospitalization, compared with only 1.4% of patients without acute renal failure. Among hospital survivors with acute renal failure, 1 and 5 year estimated mortality rates were 12.1% and 44.6%, respectively; much greater than the 3.7% and 14.5% mortality rates in patients without acute renal failure (Rihal *et al.*, 2002).

### 4.1 Prevention and Prophylaxis

**Figure 1:**
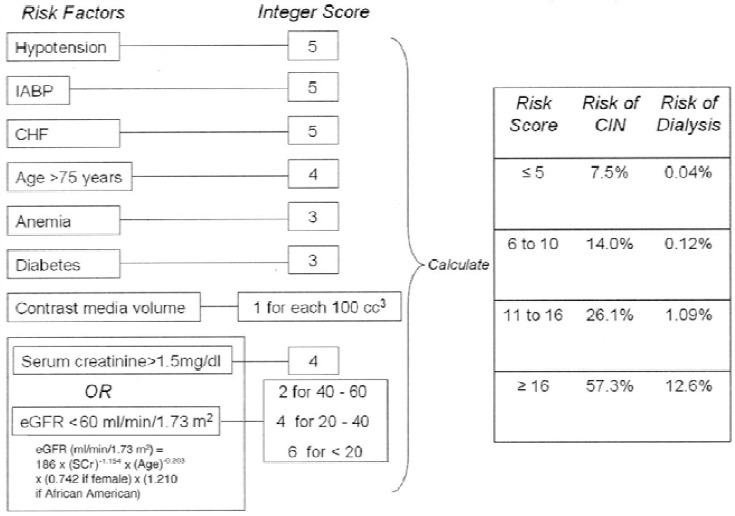
Multivariable CIN risk score (Mehran *et al*., 2004)

Multiple individual risk factors have been reported for the development of CIN. Using multivariable regression models, risk scores have been developed that can assess the risk of CIN (Figure 1) (James *et al*.; Mehran *et al.*, 2004). Of the modifiable variables, minimizing the volume of contrast medium administered is a primary defense against CIN. Radiocontrast dose was the most powerful independent predictor of nephropathy requiring dialysis (Cigarroa, Lange, Williams, & Hillis, 1989; Marenzi *et al.*, 2009; McCullough *et al.*, 1997; Rudnick *et al.*, 1997). The overall volume appears to be more relevant in patients with baseline chronic kidney disease, who have a 5-10 fold increase in CIN when more than 125-140 ml of contrast is administered, irrespective of other preventive measures (McCullough *et al.*, 1997; Taliercio *et al.*, 1991). Therefore, most experts recommend limitation of contrast volume to 3 ml/kg.

As previously discussed, the osmolality and ionic content of the selected contrast media have been closely related to a number of adverse reactions, including CIN (Barrett & Carlisle, 1993; Jo *et al.*, 2006; Lautin *et al.*, 1991; McCullough *et al.*, 2006; Rudnick *et al.*, 1995). Aspelin *et al*. showed that the iso-osmolar nonionic Iodixanol (Visipaque) reduced the relative risk of CIN by 23% when compared to low-osmolar nonionic agent iohexol (Omnipaque) (Aspelin *et al.*, 2003). The concept of osmolality being the sole contributor to CIN has been recently disputed by a randomized, double-blinded trial. The results of the CARE study failed to show any difference in CIN defined by multiple definitions following administration of non-ionic, low-osmolar Iopamidol in comparison to Iodixanol in high risk patients with or without diabetes mellitus (R. J. Solomon *et al.*, 2007). A meta-analysis, conducted by the same investigator, also has shown little difference between agents whose osmolality is < 800 mOsm/kg. Such data suggest that factors such as viscosity and ionic content, in addition to the osmolality of the agent chosen, contribute to the overall risk of developing CIN (R. Solomon, 2005).

Volume expansion is the cornerstone for prevention of CIN. The effectiveness of saline administration is well documented by a series of small observational and randomized trials. The first controlled study to explore this relationship was performed in 1994 and showed that administration of 0.45% saline alone over 24 hours was more effective than the combination of volume supplementation and diuresis with furosemide or mannitol (R. Solomon, Werner, Mann, D’Elia, & Silva, 1994). Mueller *et al*. addressed the tonicity of fluids in 1,383 patients, comparing 0.45% saline with 0.9% saline. The rate of CIN was greater in patients receiving 0.45% saline (2.0 vs 0.7%, p = 0.04), without any difference in outcomes for dialysis or length of stay (Mueller *et al.*, 2002). Subsequent to this study, a series of underpowered randomized trials have shown a moderate, but consistent benefit of isotonic saline administration at a rate of 1 ml/kg over a 24 hour period, beginning 12 hours prior to the procedure (Bader *et al.*, 2004; Krasuski, Beard, Geoghagan, Thompson, & Guidera, 2003; Weisbord & Palevsky, 2008). The success of peri-procedural hydration can be extended to patients with chronic renal failure through continuous veno-venous hemofiltration. Hemofiltration allows for administration of large volumes of fluid without the associated risk of fluid overload. In randomized trials, hemofiltration vs. standard therapy in patients with moderate to severe renal insufficiency (baseline creatinine 3.0 mg/dl) lowered the absolute need for hemodialysis by 18%, with additional reductions in in-hospital events and one-year mortality (10% vs. 30% for controls) (Marenzi *et al.*, 2003). Prophylactic hemodialysis, however, has not shown the same benefits (Vogt *et al.*, 2001).

The antioxidant agent acetylcysteine, 600-1200 mg orally before and 600 mg twice daily after the procedure for 24-48 hours, has shown inconsistent benefit in prevention of CIN (Briguori *et al.*, 2007; Coyle *et al.*, 2006; Diaz-Sandoval, Kosowsky, & Losordo, 2002; Fung *et al.*, 2004; Marenzi *et al.*, 2006; Tepel *et al.*, 2000; Webb *et al.*, 2004). Meta-analysis of the available data up to 2003 showed that the addition of acetylcysteine to intravenous hydration led to a 56% relative reduction in CIN in comparison to hydration alone (Birck *et al.*, 2003). Due to the cost effectiveness, feasibility of use, and benign side effect profile, many experts and institutions have advocated its routine use. A recent randomized international study in 2,303 patients from 46 hospitals across Brazil (ACT trial), however, has failed to show any benefit. In both groups, 12.7% of patients experienced CIN with similar elevations in serum creatinine and need for dialysis. This is the largest study conducted on the topic and may have answered the question on the potential benefit of acetylcysteine. (“Acetylcysteine for prevention of renal outcomes in patients undergoing coronary and peripheral vascular angiography: main results from the randomized Acetylcysteine for Contrast-induced nephropathy Trial (ACT),” 2011) Alkalinizing the urine with sodium bicarbonate infusion has been studied as an attractive mechanism to prevent CIN through attenuation of free radical formation(Brar *et al.*, 2008). The results, however, have varied and failed to show a consistent benefit among trials.(From *et al.*, 2008; Maioli *et al.*, 2008; Merten *et al.*, 2004; Recio-Mayoral *et al.*, 2007) Most of the benefit appears to have been derived from smaller studies that assessed outcomes soon after radiocontrast administration (Zoungas *et al.*, 2009). In some series, significant volume overload and heart failure have been reported after large volumes of sodium bicarbonate. Administration of ascorbic acid as an antioxidant, or the selective dopamine-1 agonist fenoldapam for promotion of renal plasma flow, has also failed to produce a consistent benefit.(Spargias *et al.*, 2004; Stone *et al.*, 2003)

## 5. Cholesterol Emboli

Cholesterol emboli are released as cholesterol crystals from friable vascular plaques. Distal embolization of cholesterol crystals after angiography, major vessel surgery, or thrombolysis causes a systemic syndrome.(Bashore & Gehrig, 2003; Kronzon & Saric) The diagnosis is suggested clinically by the appearance of discoloration of the extremities in a mottled purple pattern of livedo reticularis, or when there is digital cyanosis or gangrene, or neurological or renal involvement. Renal involvement is characteristically slowly progressing over a two to four week period following angiography. The diagnosis is confirmed through biopsy of affected tissues showing deposition of cholesterol crystals. Accompanying eosinophilia and elevated C-reactive protein are common laboratory features. The incidence reported in prospective studies is generally less than 2%.(Fukumoto, Tsutsui, Tsuchihashi, Masumoto, & Takeshita, 2003; Saklayen, Gupta, Suryaprasad, & Azmeh, 1997) Interestingly, autopsy studies have reported a much higher incidence, in range of 25-30%, indicating that many of these events are asymptomatic.(Fukumoto *et al.*, 2003; Ramirez, O’Neill, Lambert, & Bloomer, 1978) This is further supported by the discovery of plaque debris from > 50% of all guiding catheters in a prospective study of 1,000 patients (Keeley & Grines, 1998). No significant difference in the risk of atheroembolism between brachial and femoral approaches exists, suggesting that the ascending aorta is the predominant source. Major risk factors include advanced age, repeat procedures, diffuse atherosclerotic disease, and elevated pre-procedure C-reactive protein. Treatment is mostly supportive but one retrospective study reported decreased incidence of cholesterol emboli with pre-procedural use of simvastatin.(Woolfson & Lachmann, 1998) Besides statins, management with steroids and prostaglandins has not resulted in significant benefit.(Elinav, Chajek-Shaul, & Stern, 2002; Graziani, Santostasi, Angelini, & Badalamenti, 2001)

## 6. Local Vascular Injury

Vascular access site complications are among the most common and dreaded complications of coronary angiography, and are the most significant contributor to morbidity and mortality of the procedure. In the earlier days of cardiac catheterization, the incidence of vascular complications was reported to be between 0.7%-11.7% (Babu, Piccorelli, Shah, Stein, & Clauss, 1989; Omoigui *et al.*, 1995; Oweida, Roubin, Smith, & Salam, 1990; Samal & White, 2002; Wyman *et al.*, 1988). Over the past decade there have been significant advances in anticoagulant and antiplatelet therapies that have decreased the incidence of major cardiovascular events at a risk of increased bleeding. Major post-procedural bleeding and blood transfusions are associated with increased length of stay and decreased long-term survival. (Doyle *et al.*, 2008) Fortunately, increasing experience and strategies aimed at decreasing the risk of access site complications has paralleled improvements in pharmacotherapy. Increasing awareness of the significance of peri-procedural bleeding to overall morbidity and mortality has resulted in the development and validation of scores aimed at identifying patients that are at the highest risk of bleeding. (Applegate *et al.*, 2006; Kinnaird *et al.*, 2003; Mandak *et al.*, 1998; Nikolsky *et al.*, 2007) Analysis from the IMPACT II trial has identified modifiable risk factors, such as early sheath removal, avoiding placement of venous sheaths, and careful monitoring of heparin doses as potential ways of decreasing bleeding risk and complications.(Mandak *et al.*, 1998) Increasing experience with these complications has allowed for heightened awareness along with earlier detection and treatment techniques. Attempts at optimizing vascular access via fluoroscopic delineation of anatomical landmarks and identifying potential complications via peripheral angiography have come into routine practice (Turi, 2005). Advances in equipment design allow use of lower profile catheters via smaller sheaths, decreasing vascular trauma, and causing fewer complications.(Applegate *et al.*, 2008; Metz *et al.*, 1997; Talley, Mauldin, & Becker, 1995) The development of vascular closure devices has improved patient comfort following the procedure and, as development progresses further, could reduce the incidence of bleeding complications. Accordingly, these advances have resulted in significant decrease in vascular complications from 1998 to 2007. (diagnostic catheterization from 1.7% to 0.2%, percutaneous coronary intervention from 3.1% to 1.0%, respectively) (c).(Applegate *et al.*, 2008)

**Figure 2:**
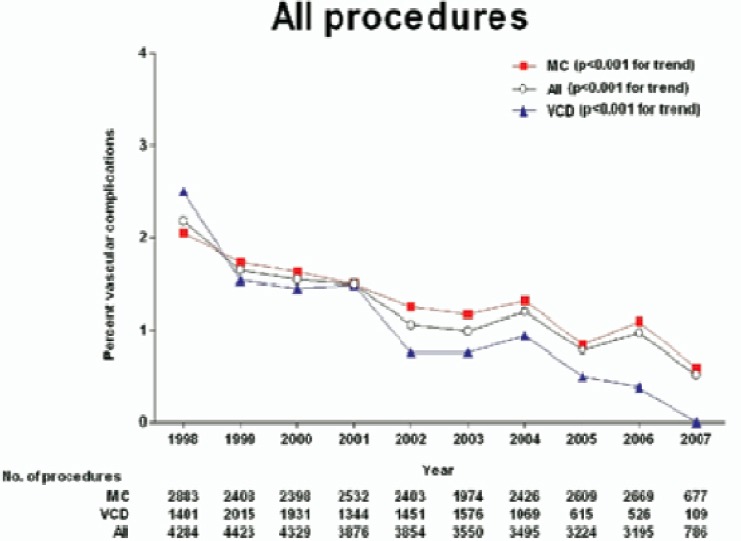
Any vascular complications by procedure and closure method. CATH - diagnostic cardiac catheterization; MC - manual compression; PCI - percutaneous coronary intervention; VCD - vascular closure device.(Applegate *et al*., 2008)

**Figure 3:**
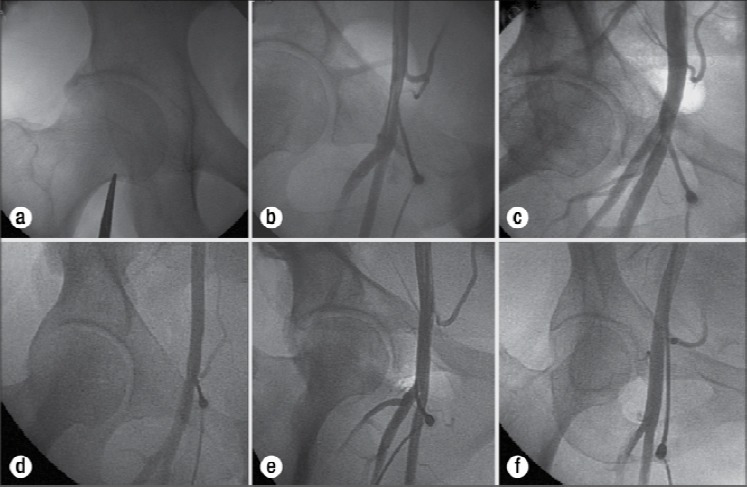
(a) Fluoroscopy of the femoral head utilizing forceps to note the position of the inferior border of the femoral head on the patient’s skin. (b) Correct placement of the sheath in the common femoral artery. (c) Correct placement of the sheath in relation to the femoral head, with the arterial access incorrectly placed in the superficial femoral artery due to the anatomic variant of a high bifurcation. (d) Correct placement of the sheath in relation to the femoral head with a low hypogastric artery causing incorrect arterial placement in the external iliac artery. (e) Low sheath placement in the profunda femoris artery. (f) High sheath placement in the external iliac artery (Jacobi *et al*., 2009).

Most of the local vascular complications can be avoided with optimal placement of the sheath in the common femoral artery (Figure 3). The common femoral artery courses over the femoral head in 92% of cases, and 99% of the time the bifurcation of the common femoral artery was below the middle of the femoral head (Garrett, Eckart, Bauch, Thompson, & Stajduhar, 2005), (Jacobi, Schussler, & Johnson, 2009; Sherev, Shaw, & Brent, 2005),(Kim *et al.*, 1992)

### 6.1 Hematoma and Retroperitoneal Hemorrhage

Poorly controlled hemostasis following femoral sheath removal can result in a self-limited collection of blood in the anterior compartment of the thigh forming a hematoma. Most hematomas are benign, tender masses without connection to the accessed vessel. Larger hematomas, however, have been associated with formation of deep vein thrombosis and nerve compression resulting in sensory loss.(Butler & Webster, 2002; Shammas, Reeves, & Mehta, 1993) Large hematomas requiring blood transfusion occurred in 2.8% of the population in a large single center registry from 2000-2005 (Table 2).(Doyle *et al.*, 2008) Manual compression of the proximal femoral artery and hematoma immediately following discovery and examination should be initiated. From our experience, 20-30 minutes of manual compression results in resolution of the hematoma when no further bleeding or false aneurysm are present. Prompt removal of access sheaths with 2-4 hours of bed rest following removal can help to decrease the incidence of femoral hematomas.

**Table 2:** Changing incidence of major femoral bleeding and blood transfusions after PCI. (*p < 0.005 versus 2000-2005)

	1994-1995 (n = 2,441)	1996-1999 (n = 6,207)	2000-2005 (n = 9,253)
**Femoral Hematoma**	172 (7.0%)*	236 (3.8%)*	257 (2.8%)

**Femoral Bleed**	60 (2.5%)*	76 (1.2%)*	54 (0.6%)

**Retroperitoneal Bleed**	20 (0.8%)*	19 (0.3%)	26 (0.3%)

**Blood Transfusion**	207 (8.5%)*	482 (7.8%)*	516 (5.6%)

**1 to 2 Units**	98 (4.0%)	288 (4.6%)*	347 (3.8%)

**3 + Units**	109 (4.5%)*	194 (3.1%)*	169 (1.8%)

Larger or rapidly expanding hematomas can lead to hemodynamic compromise and multiple blood transfusions. In this setting, free femoral bleeding secondary to laceration of the femoral artery should be suspected. In such cases, a crossover sheath should be inserted into the contralateral femoral artery and bleeding site localized via angiography. In the case of uncontained bleeding, blood loss can be controlled by inflating a peripheral angioplasty balloon or deploying a graft stent at the site of vessel trauma (Samal & White, 2002).

**Figure 4:**
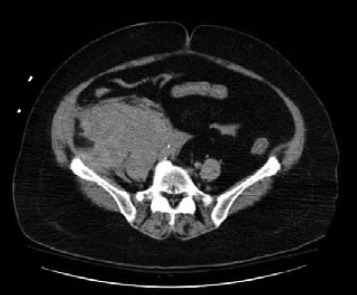
Retroperitoneal bleeding following cardiac catheterization via right femoral access.

Retroperitoneal bleeding is a potentially life threatening complication of arterial access, more frequent when the artery is punctured above the inguinal ligament. Such bleeding is typically not evident from the surface, but should be suspected when patient develops abdominal or flank pain along with hypotension and decreasing hemoglobin level. CT scans can be used to confirm clinical suspicion, but early recognition is essential in order to expedite application of manual compression and administration of fluids (Figure 4). Older age, female gender, low body surface area, and higher femoral artery puncture are significant risk factors for retroperitoneal hematomas. (Farouque *et al.*, 2005; Sherev *et al.*, 2005) Although there have been some concerns with increased risk with PCI in the glycoprotein IIb/IIIa inhibition era, no associations have been described in large retrospective studies. (Farouque *et al.*, 2005) Most patients can be managed with reversal of anticoagulation, compression of the access site, observation, and volume expansion with or without blood products. When conservative management has failed, tamponading the puncture site via balloon angioplasty from the ipsilateral or contralateral femoral artery can be successfully performed (Samal & White, 2002).

### 6.2 Pseudoaneurysm

False aneurysms develop when a hematoma maintains continuity with the arterial lumen, resulting in blood flow into and out of the hematoma cavity during systole and diastole. The origin of the nomenclature stems from lack of normal arterial wall structures (media and adventitia) despite the typical aneurysmal appearance. The incidence is between 0.5-2.0% after diagnostic angiography and has been reported in as many as 7.7% of patients when coronary or peripheral interventions are performed. (Hessel, Adams, & Abrams, 1981; Katzenschlager *et al.*, 1995) The principal risk factors for its development are similar to those for hematoma. False aneurysms occur more commonly following low access, where the superficial femoral artery is more likely to be accessed instead of the common femoral artery. This artery is smaller than the common femoral artery, making sheath insertion more traumatic. Furthermore, the lack of underlying bone provides less support for manual compression; too brief a period of manual compression is also a risk factor for its development.(Katzenschlager *et al.*, 1995) Clinically, it is detected as a pulsatile mass with bruit adjacent to the site of femoral access. The diagnosis can be made radiographically demonstrating an aneurysmal structure with a thin neck or sinus tract that connects to the femoral artery. Although angiography and contrast CT can be used to make the diagnosis, Doppler color flow imaging has been shown to be the most effective technique for identification of vascular complications (Figure 4a) (Sheikh *et al.*, 1989). Prompt diagnosis can avoid the catastrophic risk of rupture, which is likely to occur in larger aneurysms (> 3 cm), in the presence of symptoms, large hematoma, or continued growth of the sac.(Kent *et al.*, 1993; Kresowik *et al.*, 1991; Webber, Jang, Gustavson, & Olin, 2007)

**Figure 5:**
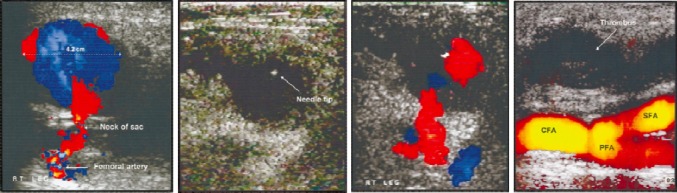
Duplex ultrasound image of pseudoaneurysm, demonstrating arterial flow through a long, narrow neck arising from defect in femoral artery and turbulent color flow into cavity (a). With color flow removed, exact position of needle tip can be identified at all times during procedure, because a small amount of echogenic thrombus forms at needle tip when thrombin comes into contact with blood, helping to guide needle placement (b). With needle in position, color flow during injection of thrombin confirms acute development of thrombus within sac (c). Power Doppler image of patent native fem- oral vessels (CFA indicates common femoral artery; SFA, superficial femoral artery; and PFA, profunda femoris artery) and absence of flow after successful thrombin injection into pseudoaneurysm cavity (d) (Lennox *et al*., 1999).

The treatment depends on the size of the false aneurysm and the rate of growth. False aneurysms less than 2-3 cm in greatest diameter can be managed expectantly and followed on an outpatient basis with serial ultrasound examinations (Johns, Pupa, & Bailey, 1991; Kent *et al.*, 1993; Kresowik *et al.*, 1991). However, aneurysmal size is not an absolute predictor of thrombosis(Kent *et al.*, 1993); therefore, patients with false aneurysms of any size should be followed closely until thrombosis occurs. Most experts advocate for intervention in symptomatic patients who have a false aneurysm > 2.0 cm (Webber *et al.*, 2007). Larger aneurysms have traditionally been repaired surgically or through ultrasound-guided compression of the femoral neck without compromising femoral artery flow (Samal & White, 2002). More recently, percutaneous injection of thrombin (1000 US U/mL) has been demonstrated as an effective method of thrombosis, with up to 96% primary success rate (Krueger *et al.*, 2005; Webber *et al.*, 2007). Ultrasound guided injection of thrombin can be completed in several minutes, has the advantage of avoiding surgical intervention or the pain associated with ultrasound-guided compression, and can be performed effectively in patients who have received anticoagulation (Figure 5a-d) (Lennox *et al.*, 1999; Pezzullo, Dupuy, & Cronan, 2000; Taylor *et al.*, 1999). Surgical repair of false aneurysms is reserved for cases which exhibit rapid expansion, infection, or failure of closure via thrombin injection. (Samal, White, Collins, Ramee, & Jenkins, 2001; Webber *et al.*, 2007)

### 6.3 Arteriovenous Fistula

**Figure 6:**
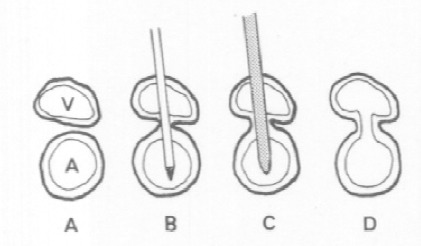
AVF result when needle tract crossing both artery and vein is dilated and catheterized. V = vein, A = artery(Kim *et al*., 1992)

Arteriovenous Fistulas (AVF) arise when a needle tract crosses both the artery and vein, with subsequent dilation during sheath insertion (Figure 6). They can also arise from on-going bleeding from the puncture site that compresses into an adjacent femoral vein. As such, they are typically caused by low arterial access into the superficial femoral artery because of the anterior-to-posterior relationship of the artery to the superficial femoral vein, as opposed to the side-by-side relationship of the common femoral artery and vein (Kim *et al.*, 1992). Diagnosis is made by the auscultation of a thrill or continuous bruit over the puncture site, and confirmed by contrast CT or Doppler sonography. Prospective follow-up studies in over 10,000 patients undergoing transfemoral cardiac catheterization have shown an incidence of almost 1%. Management is usually conservative with close follow-up, as nearly one-third of AVF closed spontaneously within one year(Kelm *et al.*, 2002) Surgical management is reserved for symptomatic patients, high output heart failure, or fistulas that do not close spontaneously within one year(Samal & White, 2002)

### 6.4 Dissection

Dissection of the femoral and iliac arteries occurs very infrequently, (0.42% of the most current cohorts). (Prasad *et al.*, 2008) It occurs more commonly in iliac arteries with increased atherosclerotic burden, tortuosity, or traumatic sheath placement. Occlusive dissection is a potentially limb and life-threatening complications that can be identified and treated safely upon diagnosis. Cine images of the femoral access site prior to completion of the study are helpful in localizing potential dissections, and should be performed in patients with difficult access or traumatic sheath placement. When detected, removal of the wires and catheters can allow for spontaneous resolution(Samal & White, 2002) When large, flow limiting dissections occur, angioplasty and stenting have been shown to be a safe and effective method of treatment and surgery can usually be avoided(Scheinert *et al.*, 2000)

### 6.5 Thrombosis and Embolism

Thrombosis typically occurs in female patients with small vessel lumen, peripheral arterial disease, diabetes mellitus, placement of a large diameter catheter or sheath (intraaortic balloon pump), or long catheter dwell-time (Noto *et al.*, 1991; Popma *et al.*, 1993). Patients typically complain of a painful leg with impaired sensation and motor function in the distal extremity. Loss of peripheral pulses and the appearance of a white painful foot can often be found on physical examination. Thrombotic and embolic complications can be avoided with careful, frequent flushing of the arterial sheaths (prevents thrombus formation) and use of anticoagulation during prolonged procedures and intraaortic balloon pump use. Treatment involves percutaneous thrombectomy or thrombolytic therapy in conjunction with vascular surgery consultation (Samal & White, 2002).

### 6.6 Vascular Closure Devices

Various methods for percutaneous closure of the femoral artery have been explored over the years in order to avoid manual compression and shorten bed rest, two of the biggest sources of dissatisfaction to patients. Although these devices allow for greater comfort and earlier ambulation, their safety and cost effectiveness in comparison to manual compression has not been unequivocally confirmed. Their benefit has been marginal, at best, and many studies have reported increased incidence of vascular complications following PCI (Koreny, Riedmuller, Nikfardjam, Siostrzonek, & Mullner, 2004; Nikolsky *et al.*, 2004). These reviews, however, were of trials of vascular closure devices in their early years of use. Technological improvements to devices and increased operator experience are likely to contribute to improved efficacy and safety. This was demonstrated in the analysis of the ACUITY trial by Sanborn *et al.*, where interventional or surgical correction or hematoma ≥ 5 cm occurred significantly less often in patients undergoing PCI when a vascular closure device was used (0.4% versus 0.8%, *P*<0.05 and 1.9% versus 2.7%, *P*<0.03, respectively) (Sanborn *et al*.). The magnitude of these reductions is very similar to those recently reported from the large Northern New England PCI Registry in which the relative risk reduction in bleeding and vascular complications with bivalirudin and vascular closure device use during PCI was 52% and 25%, respectively.(Ahmed *et al.*, 2009) Therefore, marked reductions in femoral access complications may be realized with advancement in vascular closure device technology and increased operator experience, along with adjunctive medications that decrease the risk of bleeding.

### 6.7 Transradial Approach

Transradial approach has gained progressive acceptance since its introduction over 20 years ago, largely due to decrease in vascular complications and patient satisfaction with the procedure. The approach has several advantages, in that, the radial artery is not immediately associated with nearby nerves or veins and is easily compressible, allowing for improved hemostasis. Furthermore, the hand receives dual blood supply through the ulnar and radial arteries via the palmar arch. Therefore, any radial artery occlusion (reported in 5-19%) (Greenwood *et al.*, 2005) is not clinically important in most patients because the hand is perfused by extensive collateral flow between the two arteries. When performed properly, the Allen test is an easy and effective method to assess the adequacy of collateral blood flow into the hand. In patients in whom the Allen test fails, there is an increased incidence of complications and occlusion of flow to the hand.

In a meta-analysis by Agostini *et al*. in 2004, similar rates of major adverse cardiovascular events with both access routes were observed, with significantly lower rate of entry site complications in the radial access group. The advantages are, however, balanced by the higher proportion of procedural failures, 7.2% versus 2.4% for femoral access (Agostoni *et al.*, 2004). Data from the National Cardiovascular Data Registry from 2004-2007 has shown that the proportion of radial artery interventions has been increasing steadily. However, they still only constitute 1.3% of all total procedures performed during that time. Compared with the femoral approach, use of radial PCI in recent data is associated with similar rate of procedural success but lower risk of procedural bleeding(Rao *et al.*, 2008) Increased operator experience, further development of low profile catheters and stents, along with greater patient satisfaction and comfort, fuel the interest in this field.

## 7. Conduction Disturbances

### 7.1 Bradyarrhythmia

Transient bradycardia is a common occurrence in the catheterization laboratory. They were far more frequent in the era of high osmolar ionic contrast agents, but have declined recently due to the widespread use of iso-osmolar, non-ionic contrast material. Prolonged episodes of bradycardia can lead to vagal response with associated hypotension, nausea, sweating, and yawning. This was observed in nearly 3.5% of patients in one study, 80% of which developed during access and 16% during sheath removal (Landau, Lange, Glamann, Willard, & Hillis, 1994). Treatment of anxiety and pain, along with adequate hydration can help avoid prolonged vagal reactions. Furthermore, hypotension and bradycardia can be one of the first signs of perforation and tamponade, as a vagal response is induced through irritation of the pericardium. Coughing forcefully can help to increase coronary perfusion and restore normal cardiac rhythm. When coughing is unsuccessful, rapid intravenous fluid administration, treatment of underlying pain or anxiety, and 0.5-1 mg of atropine intravenously can help reverse the bradycardia. In cases of complete heart block, temporary pacing through a transvenous pacemaker should be rapidly initiated.

Conduction disturbances also occur, but at a much lower frequency than vagal episodes. Passing of the catheter across the aortic valve will usually cause some ectopy. However, in a patient with pre-existing right bundle branch block, the development of left bundle branch block because of septal scraping can lead to complete heart block and cardiovascular collapse. Conversely, in a patient with a pre-existing left bundle branch block, right heart catheterization and right bundle branch block can cause a similar scenario. As such, the electrocardiogram of every patient should be reviewed prior to procedure by the operator. Minimizing the period of ectopy can help in avoiding these complications.

### 7.2 Tachyarrhythmia

Atrial arrhythmias may occur following irritation of the right atrium by the catheter during right heart catheterization. These arrhythmias usually do not require immediate treatment unless they produce ischemia or hemodynamic instability. The occurrence of ventricular tachycardia and ventricular fibrillation in the current era is related to irritation of the myocardium by the catheter. Identification of ventricular ectopy by trained technicians and engaged operators can help reduce the incidence of these arrhythmias. When a run of ventricular tachycardia is noted, the offending catheter must be pulled back immediately to allow restoration of normal sinus rhythm. Ventricular arrhythmias were more prominent in the era of high osmolar, ionic contrast when intracoronary injection into the right coronary artery caused ventricular dysrhythmia in 1.3% of patients.(Adams, Fraser, & Abrams, 1973; Zukerman *et al.*, 1987) The most recent reports, however, place this complication rate at 0.1% (Chen, Gao, & Yao, 2008). In patients with acute myocardial infarction, ventricular tachycardia occurred in 4.3% of the patients with ST- elevation MI during cardiac catheterization in the PAMI trial.(Mehta *et al.*, 2004) Pre-treatment of high risk patients with beta-blockers, or initiation of anitarrhythmic therapy with lidocaine or amiodarone during recurrent episodes should be considered as treatment options. Hemodynamically unstable atrial rhythms or any sustained ventricular tachyarrhythmia should be treated with direct current cardioversion.

## 8. Death

During the last few decades, the incidence of death has progressively declined during left heart catheterization. In the early 1960s, 1% mortality was observed with diagnostic catheterization, which has decreased to 0.08% in the 1990s(Braunwald & Gorlin, 1968; Chandrasekar *et al.*, 2001; Johnson *et al.*, 1989; Kennedy, 1982; Noto *et al.*, 1991) There are a number of baseline variables which contribute to mortality during coronary angiography: presence of multivessel disease, left main coronary artery disease (LMCA), CHF, renal insufficiency and advanced age are the most important of them (Laskey, Boyle, & Johnson, 1993). In recent years, cardiac catheterization and percutaneous coronary intervention have witnessed new developments, such as stents and potent antiplatelet agents that could affect the overall complication rate.(Chandrasekar *et al.*, 2001)

Significant LMCA disease increases the risk of dissection during catheter engagement and injection of contrast, which is reported to be around 0.07% and almost twice higher with percutaneous intervention(Cheng *et al.*, 2008; Eshtehardi *et al*.) Mortality associated with iatrogenic LMCA dissection is reported around 3% (Eshtehardi *et al*.), particularly if undetected. Emergency therapeutic interventions with either coronary artery bypass surgery or percutaneous coronary intervention with stents are required.

Patient with depressed chronic left ventricular function and those with acute MI who are in shock are at the highest risk for mortality.(Anderson *et al.*, 2007; Johnson *et al.*, 1989; Shaw *et al.*, 2002) If frank cardiogenic shock is present or develops during cardiac catheterization, intra-aortic balloon pump and inotropic support may be required.

If PCI is performed in addition to coronary angiography, the incidence of mortality is higher.(Dorros *et al.*, 1983; Shaw *et al.*, 2002) Recent data from the American College of Cardiology-National Cardiovascular Data Registry (ACC-NCDR) published in 2010 have shown that factors which are associated with increased risk of mortality during PCI are cardiogenic shock, increasing age, salvage, urgent or emergency PCI, decreased left ventricular ejection fraction, acute MI, diabetes, chronic renal failure, multivessel disease, prior coronary artery bypass grafts (CABG) and chronic occlusion. Overall, PCI in-hospital mortality was 1.27%, ranging from 0.65% in elective PCI to 4.81% in ST- elevation MI patients. (Anderson *et al.*, 2007; Peterson *et al*.; Shaw *et al.*, 2002)

Patients with aortic stenosis have higher mortality; the VA Cooperative study on Valvular Heart Disease has shown a 0.2% mortality among 1559 preoperative catheterizations. (Folland *et al.*, 1989) Bartsch *et al*. have shown a mortality rate 1.1% in patients with aortic value stenosis requiring left heart catheterization to determine the transvalvular gradient.(Bartsch, Haase, Voelker, Schobel, & Karsch, 1999)

Patients with history of CABG who required diagnostic and therapeutic cardiac catheterization are typically older and have generalized atherosclerosis, worse left ventricular function, and require a more lengthy and complex procedure. Varghese *et al*. have shown that patients with coronary artery bypass undergoing graft PCI have no difference in terms of mortality as compared to patients with CABG undergoing native vessel PCI (Varghese, Samuel, Banerjee, & Brilakis, 2009),(Garcia-Tejada *et al.*, 2009).

## 9. Myocardial Infarction

Myocardial damage can occur in different clinical settings: spontaneous, during diagnostic cardiac catheterizations, during percutaneous intervention and during CABG surgery. There have been different thresholds for identifying an infarct in clinical trials: CK-MB > 2 times the upper limit of normal (ULN) for spontaneous MI; CK-MB > 3 times the ULN with coronary interventions; and CK-MB > 5-10 times the ULN for bypass surgery (Alpert, Thygesen, Antman, & Bassand, 2000). In the late 1970s, the data from the Coronary Artery Surgery Study (CASS) showed a MI rate of 0.25% for coronary angiography (Davis *et al.*, 1979). In the first, second, and third registries conducted by the Society for Cardiac Angiography, the risk of MI fell progressively, from 0.07%, to 0.06%, to 0.05% (Johnson *et al.*, 1989; Kennedy, 1982; Noto *et al.*, 1991). The risk of MI during diagnostic catheterization is clearly influenced by patient-related factors that include the extent of CAD (0.06% for single vessel disease, 0.08% for triple-vessel disease, and 0.17% for left main disease) (Johnson *et al.*, 1989). With improvement in the equipment and operator skill, the use of more potent antithrombotic and antiplatelet agents, better patient preparation with the use of beta blockers and statins and adoption of low osmolar contrast agents, the incidence of myocardial MI during cardiac catheterization has been reduced considerably (Judkin & Gander, 1974; Pasceri *et al.*, 2004).

Approximately 1.5 million patients undergo PCI in the United States every year (Roger *et al*.). Depending on local practices and diagnostic criteria used, 5 to 30% of these patients have evidence of peri-procedural MI.

At the higher estimate, the incidence of these events is similar to the annual rate of major spontaneous MI. The predictors of peri-procedural MI can be broadly categorized as patient-, lesion-, and procedure- related risk factors (Herrmann, 2005). The major risk factors in terms of both frequency and extent, are complex lesions (e.g., the presence of thrombus, stenosis of a saphenous-vein graft, or a type C lesion), complex procedures (e.g., treatment of multiple lesions or use of rotational atherectomy), and procedural complications (e.g., abrupt vessel closure, side-branch occlusion, distal embolization, or no reflow). (Herrmann, 2005; Mandadi *et al.*, 2004; van Gaal *et al.*, 2009) The occurrence of peri-procedural ischemic symptoms, particularly chest pain at the end of the procedure, or electrocardiographic evidence of ischemia defines the sub-group of patients most likely to have peri-procedural MI (Cai *et al.*, 2007).

Large peri-procedural myocardial MIs are usually due to angiographically visible complications; however, this is generally not the case in the vast majority of patients with elevated biomarker levels after PCI. Cardiac magnetic resonance imaging has confirmed two distinct locations for peri-procedural myonecrosis: adjacent to the site of the intervention, where the injury is most likely due to epicardial side-branch occlusion and downstream from the intervention site, where it is most likely due to compromise of the microvascular circulation.

Studies evaluating the relationship between the post-procedural cardiac troponin level and long term mortality, in general, have not excluded patients with acute coronary syndromes (ACS), many of whom had abnormal cardiac biomarker levels at baseline. (Cantor *et al.*, 2002; Cavallini *et al.*, 2005; D. N. Feldman, Minutello, Bergman, Moussa, & Wong, 2009; Kini *et al.*, 2004; Kizer *et al.*, 2003; Nallamothu *et al.*, 2003; Natarajan *et al.*, 2004; Nienhuis, Ottervanger, Bilo, Dikkeschei, & Zijlstra, 2008; Testa *et al.*, 2009) Thus, the reported frequency of post-procedural elevations in cardiac troponin has been highly variable, and although some studies showed that the serum concentration of cardiac troponin was an independent predictor of survival, others did not.

It remains uncertain whether a similar amount of damage in different settings has the same prognostic implications. Mahaffey *et al*. have studied the outcome of peri-procedural MI versus spontaneous MI in a large pool of 16,173 patients from PURSUIT and PARAGON B trials of non-ST- elevation MI. It was clearly evident that patients with peri-procedural or spontaneous MI had significantly higher one and six month mortality. (Mahaffey *et al.*, 2005) A recent analysis from the ACUITY trial was conducted among 7,773 patients with moderate to high risk, non-ST elevation MI who underwent PCI. (Prasad *et al.*, 2009) Peri-procedural and spontaneous MIs during follow-up developed in 6.0% and 2.6% of the cohort, respectively. After adjustment for differences in baseline and procedural characteristics, spontaneous MI was a powerful independent predictor of an increased risk of death, whereas peri-procedural MI was not significantly associated with an increased risk of death. Similar observation was made among patients with diabetes and stable CAD in Bypass Angioplasty Revascularization Investigation 2 Diabetes (BARI 2D) trial. (Chaitman *et al.*, 2009)

Taken together, contemporary studies indicate that spontaneous MI is a powerful predictor of mortality. Peri-procedural MI, although frequent, is a marker of atherosclerosis burden and procedural complexity, but in most cases, it does not have important independent prognostic significance in stable CAD or in non-ST-elevation MI. Although large peri-procedural infarcts may affect prognosis, they rarely occur in the absence of procedural complications or in patients with normal baseline cardiac troponin levels.

## 10. Cerebrovascular Complications

**Figure 7:**
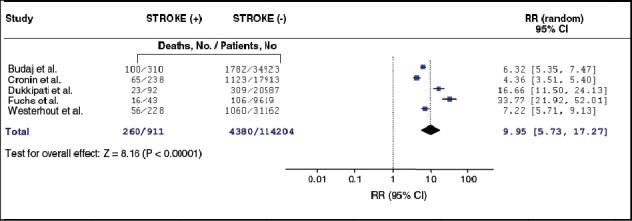
Pooled relative risk (random effects) of mortality after stroke in PCI or in patients with non ST- elevation MI

Although the overall incidence of stroke after left heart catheterization or percutaneous intervention is low, it is the most debilitating complication and is associated with a high rate of morbidity and mortality (Table 3, Figure 7) (Akkerhuis *et al.*, 2001; Fuchs *et al.*, 2002; Lazar *et al.*, 1995; Wong, Minutello, & Hong, 2005). Early experience showed an incidence as high as 0.23% in the 1973 study of Adams and others (Adams *et al.*, 1973), compared with the 0.07% incidence for the more recent diagnostic catheterization data included in the Society for Cardiac Angiography-registries (Johnson *et al.*, 1989; Kennedy, 1982).

**Table 3:** Incidence of peri-procedural stroke in PCI registries(Hamon, Baron, & Viader, 2008)

Reference	No. Patients	No.	Percentage	95% CI
**Lazar *et al*., 1995**				
**Total**	6465	27	0.42	0.27-0.60
**Ischemic**		NA	NA	NA
**Hemorrhagic**		NA	NA	NA
**Uncertain**		NA	NA	NA
**Akkerhuis *et al*., 2001**				
**Total**	8555	31	0.37	0.24-0.51
**Ischemic**		19	0.22	0.13-0.34
**Hemorrhagic**		12	1.4	0.07-0.24
**Uncertain**		1	0.01	0.00-0.06
**Fuchs *et al*., 2002**				
**Total**	9662	43	0.44	0.32-0.6
**Ischemic**		21	0.22	0.13-0.33
**Hemorrhagic**		20	0.21	0.13-0.32
**Uncertain**		2	0.01	0.00-0.07
**Dukkipati *et al*., 2004**				
**Total**	20679	92	0.44	0.36-0.54
**Ischemic**		43	0.21	0.15-0.28
**Hemorrhagic**		13	0.06	0.03-0.10
**Uncertain**		36	0.17	0.12-0.24
**Wong *et al*., 2005**				
**Total**	76903	140	0.18	0.15-0.21
**Ischemic**		NA	NA	NA
**Hemorrhagic**		NA	NA	NA
**Uncertain**		NA	NA	NA

The risk of stroke, as expected, is somewhat higher with coronary intervention, because of use of guiding catheters, multiple equipment exchanges in the aortic root, aggressive anticoagulation and longer procedure times. In 20,697 patients who underwent PCI in a large-volume center, stroke occurred in 0.44%(Dukkipati *et al.*, 2004). Multivariable analysis has shown that occurrence of stroke was associated with diabetes, hypertension, prior stroke and renal failure and was an independent predictor of in-hospital death (Hamon *et al.*, 2008). Patients who suffered a stroke had undergone longer cardiac catheterization procedures, received more contrast, were more likely to have had the procedure for urgent reasons, and to have intra-aortic balloon counter pulsation.(Stone *et al.*, 1997) Possible explanations for this latter characteristic include the greater propensity for hemodynamic compromise in these patients, which may increase the risk of ischemic stroke. Indeed, scraping of aortic plaque occurs in > 50% of PCI cases and more frequently with larger catheters (Keeley & Grines, 1998).

Cerebral micro-embolism is thought to be the main mechanism of peri-procedural ischemic stroke occurring with PCI. This finding is supported by transcranial doppler studies performed during cardiac catheterization, which show the systematic occurrence of cerebral micro-emboli.(Bladin *et al.*, 1998; Hamon *et al.*, 2006; Leclercq *et al.*, 2001) Air embolism, thrombus formation in the catheter or its surface, or dislocation of aortic atheroma during manipulation and passage of catheters within the aorta are the main sources of embolic material causing ischemic stroke during cardiac catheterization or PCI. As expected, patients with CAD more frequently have severe atheroma in the descending aorta and aortic arch than patients without CAD (Khoury, Gottlieb, Stern, & Keren, 1997).

In addition to the aortic root, embolic material may also originate in the cardiac chambers, thrombotic coronary arteries, or the surface of cardiac valves. One should avoid placing the pigtail catheter in the left ventricle in patients with suspected aneurysm or recent MI, since either condition may be associated with potentially dislodgeable mural thrombus.

Peri-procedural strokes associated with invasive procedures are predominantly (80%) attributable to embolic material that lodges in cerebral arteries. However, given the increasingly aggressive antithrombotic environment used in PCI, especially in ACS, cerebral hemorrhages are also encountered. This means that ischemic or hemorrhagic mechanisms have to be documented before any treatment can be initiated.

## 11. Dissection and Perforation of Great Vessels

**Figure 8:**
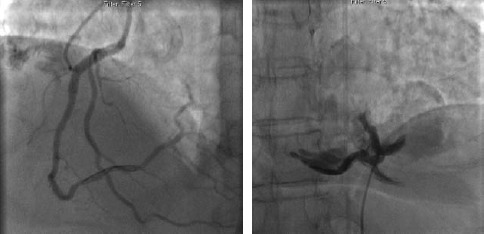
Angiogram of right coronary artery before (a) and after perforation (b)

**Figure 9:**
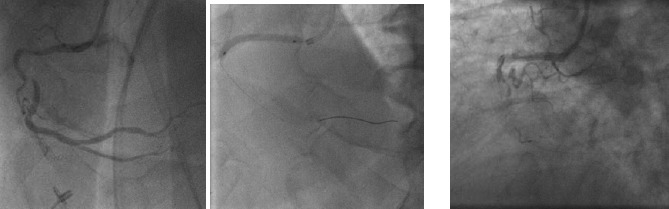
Angiogram of right coronary artery prior to intervention (a), after balloon angioplasty (b) and dissection (c)

**Figure 10:**
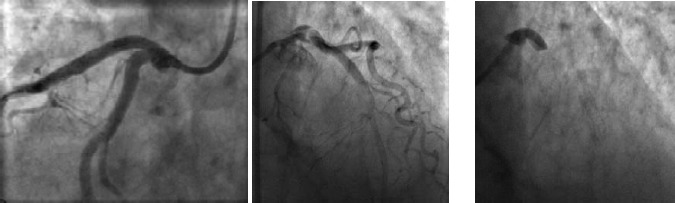
Angiogram of the left coronary system (a). Dissection of the left circumflex artery with guidewire catheter (b) with subsequent extension in to the left anterior descending artery (c)

Perforation of the cardiac chambers, coronary arteries or intrathoracic great vessels is fortunately a rare event in diagnostic catheterization. The incidence of catheter-induced ascending aorta dissection is reported around 0.04% of cases (Gomez-Moreno *et al.*, 2006). The incidence of coronary artery dissection is reported around 30% with balloon angioplasty (Figures 8-10).(Cowley, Dorros, Kelsey, Van Raden, & Detre, 1984; Huber, Mooney, Madison, & Mooney, 1991)

In recent registries the incidence of coronary artery perforation has been reported to occur in 0.3-0.6% of patients undergoing PCI. (Cowley *et al.*, 1984; Dippel *et al.*, 2001; Ellis *et al.*, 1994; Gruberg *et al.*, 2000) With the use of hydrophilic guidewires, platelet IIb/IIIa receptor blockers, and more aggressive atherectomy technologies, the incidence of coronary perforation may be higher. Some perforations, particularly those limited to deep injury to the vessel wall with localized perivascular contrast staining, can simply be observed. But such patients are at risk for delayed tamponade during the several hours following the procedure and must be monitored carefully. In contrast, free perforation may lead to development of frank tamponade within seconds to minutes, particularly when the patient is fully anticoagulated. Immediate measures should be used to seal the perforation with inflation of balloon proximal to the perforation. If after 10-15 minutes or development of ischemia, the extravasation of contrast persists, graft stents need to be utilized to seal the arterial rupture. In parallel, pericardiocentesis should be considered to provide the time necessary to seal the perforation. Overall, the reported incidence of required emergency surgery following diagnostic angiography is 0.05%, and 0.3% following therapeutic procedures (Chandrasekar *et al.*, 2001; Loubeyre *et al.*, 1999). However, once coronary artery perforation is diagnosed, the reported incidence of emergency surgery requiring pericardial window, bypass surgery or coronary artery ligation is as high as 24-40% (Table 4).

**Table 4:** Incidence of coronary artery perforation with in-hospital complications (Nair & Roguin, 2006)

Reference	Patients	Incidence	CABG	MI	Death
**Bittl *et al*., 1993**	764	3%	34.7	4.3	9
**Ajluni *et al*., 1994**	8932	0.40%	37	26	5.6
**Holmes *et al*., 1994**	2759	1.30%	36.1	16.7	4.8
**Ellis., 1994**	12900	0.50%	24	19	0
**Cohen *et al*., 1996**	2953	0.70%	41	45.5	9
**Gruberg *et al*., 2000**	30746	0.29%	39	34	10
**Dippel., 2001**	6214	0.58%	22	NA	11
**Gunning *et al*., 2002**	6245	0.80%	39	29	42
**Fejka *et al*., 2002**	25697	0.12%	39	29	42
**Stankovic *et al*., 2004**	5728	1.47%	13	27	8
**Witzke *et al*., 2004**	12658	0.30%	5	18	2.5
**Ramana *et al*., 2005**	4886	0.50%	0	20	8

Perforation of the great vessels (aorta or pulmonary artery) is extremely rare. Ascending aortic dissection can also result from vigorous use of a guiding catheter or extension from proximal coronary dissection.

Right heart catheterization can cause cardiac perforation; it is usually accompanied by bradycardia and hypotension owing to vasovagal stimulation. As blood accumulates in the pericardium, the cardiac silhouette may enlarge and the normal pulsation of the heart borders on fluoroscopy will become blunted. If the patient is hemodynamically compromised, immediate pericardiocentesis is should be performed via the subxiphoid approach. Once pericardiocentesis has stabilized the situation, the operator must decide whether or not emergency surgery will be needed to over sew the site of perforation. Most perforations, in fact, will seal spontaneously, so that surgery is unnecessary.

## 12. Other Complications

### 12.1 Hypotension

Reduction in arterial blood pressure is one of the most common problems seen during catheterization. This reduction is the final common manifestation of a variety of conditions including the following: (a) hypovolemia, owing to inadequate hydration before procedure, or excessive contrast-induced diuresis; (b) reduction in cardiac output, tamponade, arrhythmia or valvular regurgitation; or (c) inappropriate systemic arteriolar vasodilatation, due to vasodepressor response to contrast or (d) potential bleeding from retroperitoneal hemorrhage.

Low filling pressures mandate rapid volume administration, whereas low filling pressure combined with inappropriate bradycardia indicates a vasovagal reaction and atropine should be given in addition to fluid resuscitation. High filling pressures, however, suggest primary cardiac dysfunction and should prompt consideration of ischemia, tamponade, or sudden onset of valvular regurgitation. Such patients should be supported empirically by inotropic agents, vasopressors or circulatory support devices.

Patients with hypotension and normal or high cardiac output measured through saturation Swan-Ganz catheters are more likely to have an allergic reaction to contrast and may require vasopressor support, steroids and histamine blockers.

### 12.2 Hypoglycemia

Diabetic patients who are required to fast before procedure may develop hypoglycemia; special attention should be given to these patients and finger-stick blood glucose should be monitored closely before and during the procedure. If any signs of hypoglycemia, including anxiety, or lethargy develop, prompt action should be taken to administer intravenous glucose.

### 12.3 Respiratory insufficiency

Respiratory insufficiency can occur from a variety of reasons, including CHF with pulmonary edema, pre-existing lung disease, and allergic reaction or over-sedation. Immediate assessment of patient’s condition is required and therapeutic measures should be taken based on the presumed etiology.

## 13. Conclusion

Cardiac catheterization is a relatively safe procedure with few complications. Although advances in medical management and equipment design have added further significant reductions to the already low incidence of complications, operator awareness and appropriateness of response remain as the most important predictors to adverse outcomes. With each coronary angiography the potential benefit of the procedure should be weighed against the established risk factors with the well-defined morbidity and mortality. The widespread use and availability of angiography will likely fuel further advances in percutaneous modalities that may increase patient comfort while simultaneously reducing complications further.
